# Affected parent sex and severity of autosomal dominant polycystic kidney disease: a retrospective cohort study 

**DOI:** 10.5414/CN109247

**Published:** 2017-10-16

**Authors:** Kristen L. Nowak, Michel Chonchol, Zhiying You, Malika Gupta, Berenice Gitomer

**Affiliations:** University of Colorado Denver Anschutz Medical Campus, Aurora, CO, USA

**Keywords:** ADPKD, ESRD, epidemiology, hypertension, polycystic kidney disease

## Abstract

Objective: Parental inheritance may differentially affect autosomal dominant polycystic kidney disease (ADPKD) severity via genetic imprinting or in utero epigenetic modifications; however, evidence is inconsistent. We conducted a longitudinal retrospective cohort study to assess the association between sex of the affected parent and time to hypertension diagnosis, end-stage renal disease (ESRD), and death in patients with the PKD1 genotype. Materials and methods: 814 individuals who participated in research at the University of Colorado were studied. Kaplan-Meier survival analysis was performed. The predictor was parental sex, and outcomes were diagnosis of hypertension, progression to ESRD, and death. We also examined associations in four strata according to affected parent and participant sex, as previous studies have reported earlier onset of ESRD in males compared to females. Results: The median follow-up for each outcome was as follows: hypertension, 30 (interquartile range (IQR): 18, 37); ESRD, 43 (IQR: 31, 52), death 39 (IQR: 25, 52) years of age. Among affected offspring in the entire cohort, there was no difference in hypertension diagnosis (p = 0.97) or progression to ESRD (p = 0.79) according to affected parent sex; however, participants with an affected mother were more likely to die than participants with an affected father (p < 0.05). In stratified analyses, males were more likely than females to develop hypertension and reach ESRD when the affected parent was the father (p < 0.01) but not when the affected parent was the mother (p ≥ 0.11). Conclusions: Our results are largely in contrast to the hypothesis that severity of ADPKD is worse with maternal inheritance of disease.

## Introduction 

Autosomal dominant polycystic kidney disease (ADPKD) is the most common inherited renal disease, affecting ~ 1 : 500 to 1 : 1,000 live births [1] and 12.5 million world-wide [2]. It is the cause of end-stage renal disease (ESRD) in ~ 4 – 10% of ESRD patients [2]. While mutations in the *PKD1* gene, affecting ~ 70 – 75% of cases, are known to have on average a more severe disease progression than *PKD2* mutations [3, 4, 5], there is considerable variability in the course of the disease [5, 6]. 

Variability in progression and severity of APDKD may be due in part to type of mutation [5], polymorphisms of other genes that modify disease severity [7] as well as gene-environment interactions [6]. It has also been suggested that parental inheritance may differentially affect ADPKD severity via genetic imprinting or in-utero epigenetic modifications; however, evidence is inconsistent [8, 9, 10, 11, 12]. 

Genetic imprinting due to the presence of epigenetic marks in certain regions of the genome may result in differential silencing of certain genes when inherited maternally compared to paternally [13, 14]. Imprinted genes may influence both fetal development as well as birth, including development of disease in adulthood [15]. Some evidence has suggested that genetic imprinting may occur in ADPKD, with more severe disease with maternal compared to paternal inheritance [8, 9], but evidence is limited and inconsistent [10, 11, 12]. Additionally, the prenatal environment could lead to epigenetic modifications, in particular DNA methylation, that influence later phenotype and disease risk [16]. 

Accordingly, using a retrospective cohort design, we evaluated the role of parental inheritance of ADPKD upon severity of ADPKD. Specifically, we evaluated the time to endpoints of hypertension, ESRD, and death in participants with the *PKD1* genotype. We hypothesized that these endpoints would occur earlier, consistent with more severe ADPKD, in participants with maternal inheritance of disease. 

## Materials and methods 

### Study design 

Since 1985, the University of Colorado has maintained a registry of patients with ADPKD who participated in a longitudinal natural history study of ADPKD. We identified 897 participants with ADPKD and presence of the *PKD1* genotype, determined by linkage analysis, who were seen between 1985 and 2012 (or provided information about a family member during this period (some events occurred prior to 1985)) and had information available regarding sex of an affected parent. Inclusion was limited to those with a *PKD1* genotype as individuals with *PKD2* are known to have a slower disease course on average [2]. Of these 897 participants, 814 individuals had data available on at least one of the outcomes of interest (hypertension diagnosis, ESRD, or death) and were included in the present analyses. All the analysis was retrospective, and participants did not return specifically for this study. 

The study was approved by the Colorado Multiple Institutional Review Board and conforms with the Declaration of Helsinki. The nature, benefits, and risks of the study were explained to all participants, and subject or parental written informed consent was obtained prior to study enrollment. Participants consented to storage of data for future analyses at the time of consent. Children over 7 and under 18 years of age provided an assent. 

### Study variables 

**Predictor**

The predictor variable was sex of an affected parent with a known diagnosis of ADPKD. Further stratification occurred according to sex of the study participant. These data were collected by self-report or by family participation in research. 

**Outcomes**

Outcomes of interest, defined a priori, were diagnosis of hypertension, progression to ESRD, and death due to any cause. All outcomes were evaluated by clinical evaluation including detailed medical history or questionnaire asking the participant or a family member whether the outcomes had occurred, and if so, in what year [7]. 

**Other measurements**

Year of birth, sex, age at diagnosis, and very-early onset (VEO) disease were determined either by clinical evaluation or from self-report. VEO status was defined as children diagnosed either in utero or within the first 18 months of life [17, 18]. Cause of death was determined by self-report by family member and, whenever possible, confirmed by autopsy report. Cause of death was categorized as ESRD (death attributable to ESRD prior to 1975 [19] or known withdrawal from or refusal of dialysis), cardiovascular (cardiac disease or ruptured abdominal aortic aneurysm), infection, neurological event (stroke or ruptured cerebral intracranial aneurysm), cancer, trauma (car accident or suicide), gastrointestinal (gastrointestinal bleeding, perforated colon, or liver disease), in utero or infancy, pulmonary (pulmonary embolism, asthma), and other (eclampsia (n = 1), or hypothermia (n = 1)), or unknown. 

**Statistical analyses**

The longitudinal association between sex of an affected parent and severity of ADPKD (time to diagnosis of hypertension, progression to ESRD, or death), was examined by survival analyses including Kaplan-Meier, the log-rank test, and the Cox proportional hazard regression models. As we were interested in understanding differences in the proposed associations according to participant sex, we also stratified the analyses according to sex of the study participant and tested for multiplicative interactions by sex. However, irrespective of p-values, we planned a priori to explore these associations in four separate strata according to affected parent and participant sex, as previous epidemiological studies have reported earlier onset of ESRD in males compared to females with ADPKD [9, 11, 20, 21]. Sidak-adjusted p-values (for multiple comparisons of log-rank test) comparing these four strata were also evaluated. The initial time point was defined as birth (0 years of age) for all participants. Participants were censored upon cessation of participation in research. 

To better understand the impact of deaths in utero and during infancy upon the survival analysis with death as an endpoint, we performed a post-hoc sensitivity analysis excluding individuals with in-utero/infancy deaths. Survival analyses including Kaplan-Meier, the log-rank test, and the Cox proportional hazard regression models were repeated with these 4 individuals excluded. 

Differences in demographics and clinical characteristics between groups were assessed using t-tests, χ^2^-tests, or Fisher’s exact test. Unadjusted Cox proportional hazards analysis was used to determine the hazard ratio for each outcome of interest. Two-tailed values of p < 0.05 were considered statistically significant. All statistical analyses were performed with SAS version 9.4. 

## Results 

### Clinical characteristics 

Demographics and clinical characteristics according to sex of the affected parent are shown in Table 1. Participants included in the study were from 119 families, and 91% of patients were related to another study participant; however, this relationship may have been distant as many of the family trees are quite extensive. The median follow-up for each outcome was as follows: hypertension, 30 years (interquartile range (IQR): 18, 37); ESRD, 43 years (IQR: 31, 52), death 39 years of age (IQR: 25, 52). Participants with an affected mother were diagnosed at a younger age and were more likely to have VEO-ADPKD compared to those with an affected father, but they did not significantly differ in sex or reaching an endpoint of hypertension, ESRD, or death. Overall, causes of death in those who died did not differ between those with an affected mother compared to an affected father; however, all instances of in utero or infancy deaths occurred in participants with an affected mother (p = 0.06). 

### Hypertension 

**Figure 1.**
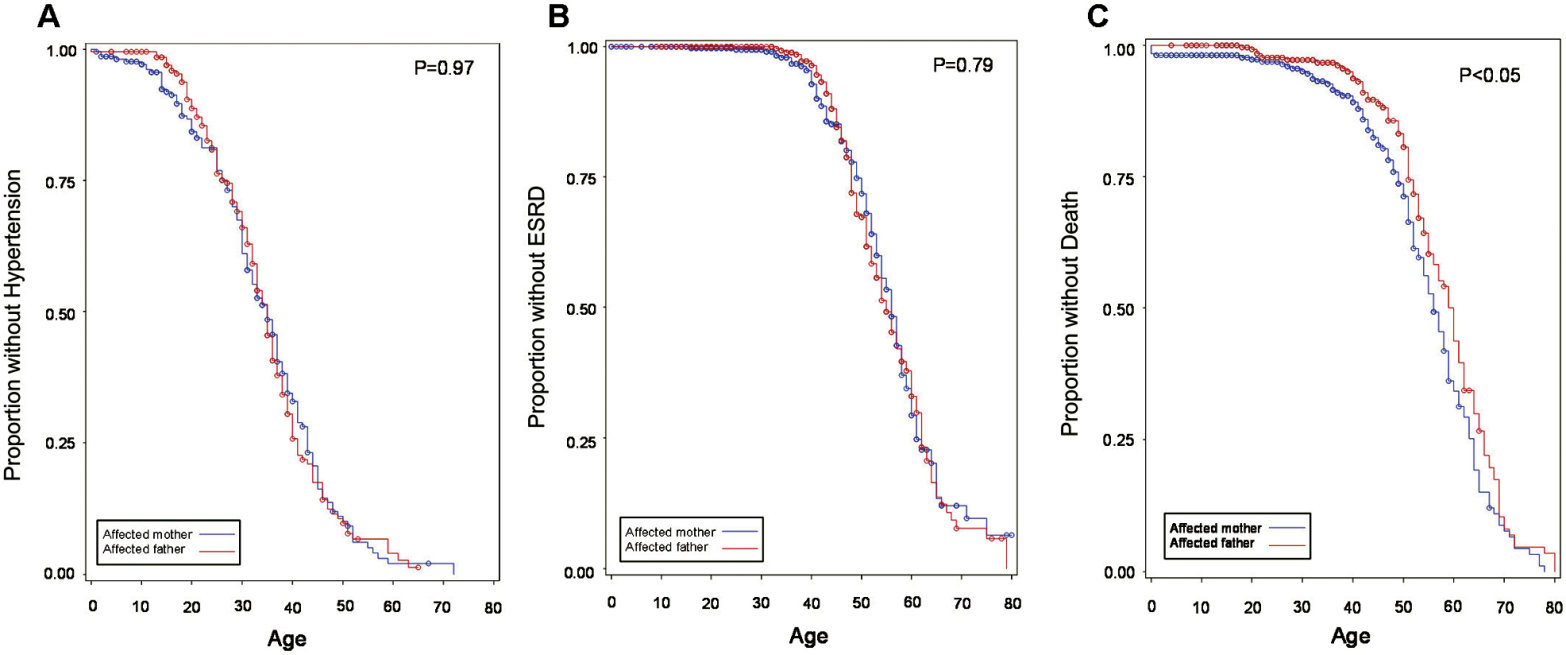
Kaplan-Meier curve of hypertension (A), end-stage renal disease (ESRD) (B), and death (C), according to sex of the affected parent. Likelihood of hypertension diagnosis or reaching ESRD did not differ according to sex of affected parent, but autosomal dominant polycystic kidney disease (ADPKD) patients with an affected mother were more likely to die than those with an affected father.

142 (65%) participants with affected mothers and 145 (70%) participants with affected fathers were diagnosed with hypertension at a known age (these numbers differ from Table 1 as some participants were diagnosed at an unknown age). Diagnosis of hypertension did not differ according to sex of the affected parent (Figure 1A) (p = 0.97). The median age at diagnosis of hypertension was similar in each group (Table 2). The hazard ratio (HR) for diagnosis of hypertension for participants with an affected mother compared to an affected father was 1.00 (95% confidence interval (CI): 0.79, 1.26). 

**Figure 2.**
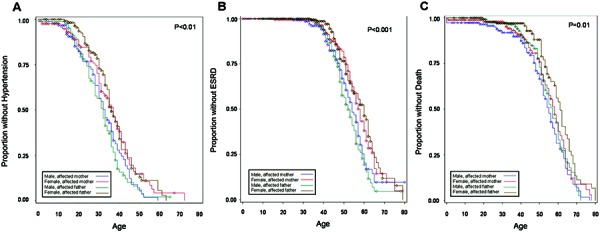
Kaplan-Meier curve of hypertension (A), end-stage renal disease (ESRD) (B), and death (C), according to sex of the affected parent and sex of the participant. Likelihood of hypertension, ESRD, and death differed according to the four strata, with the earliest hypertension diagnosis and age of ESRD occurring in males with an affected father, and death occurring earliest in males with an affected mother.

The p-value for the participant sex * affected parent sex interaction term was not statistically significant (p = 0.47). However, the four sex * affected parent strata significantly differed in time to diagnosis of hypertension (Figure 2A) (p < 0.01). Males were more likely to be diagnosed with hypertension when the affected parent was the father (p < 0.01), but there was no difference between males and females when the affected parent was the mother (p = 0.14). The median age of hypertension diagnosis ranged from 32 years (IQR: 24, 38) for males with affected father to 37 years (IQR: 48, 64) for females with an affected mother (Table 2). 

### ESRD 

131 (34%) participants with affected mothers and 133 (39%) participants with affected fathers reached ESRD at a known age. Progression to ESRD did not differ according to sex of the affected parent (Figure 1) (p = 0.79). The median age at ESRD was similar in each group (Table 2). The hazard ratio for progression to ESRD for participants with an affected mother compared to an affected father was 0.97 (95% CI: 0.76, 1.23). 

The p-value for the participant sex * affected parent sex interaction term was not statistically significant (p = 0.28). However, the four sex * affected parent strata significantly differed in time to ESRD (Figure 2) (p < 0.001). Males were more likely to reach ESRD when the affected parent was the father (p < 0.001), but there was no difference between males and females when the affected parent was the mother (p = 0.11). The median age of ESRD ranged from 52 years (IQR: 47, 59) for males with affected father to 60 years (IQR: 51, 65) for females with an affected mother (Table 2). 

### Death 

124 (39%) participants with affected mothers and 107 (36%) participants with affected fathers died at a known age. Participants with an affected mother were more likely to die due to any cause than participants with an affected father (Figure 1C) (p < 0.05). Median age of death was earlier in participants with affected mothers (56 (49, 64) years) compared to affected fathers (60 (51, 66)) (Table 2). The HR for death for participants with an affected mother compared to an affected father was 1.34 (1.03, 1.75). In sensitivity analyses excluding 4 cases of in-utero or infancy death, this HR was attenuated but remained statistically significant (HR: 1.32 (1.01, 1.72)). 

The p-value for the participant sex * affected parent sex interaction term was not statistically significant (p = 0.40). However, the four sex * affected parent strata significantly differed in time to death (Figure 2C) (p = 0.01). Males tended to be more likely than females to die when the affected parent was the father (p = 0.07) but not when the affected parent was the mother (p = 0.50). The median age of death ranged from 55 years (IQR: 48, 63) for males with an affected mother to 61 years (IQR: 53, 67) for females with an affected father to (Table 2). 

## Discussion 

In this retrospective cohort study, we found no difference in time to diagnosis of hypertension or ESRD in participants with ADPKD according to sex of the affected parent. However, participants with an affected mother were more likely to die than those with an affected father. In contrast, when stratified by participant sex, males were more likely than females to develop hypertension and reach ESRD when the affected parent was the father but not when the affected parent was the mother. 

Previous analyses of the influence of parental sex upon ADPKD severity have been limited and inconsistent in findings. One study of prognosis within 17 *PKD1* families found evidence of genetic imprinting of disease progression, with age of ESRD occurring earlier in individuals with an affected mother (51 years) compared to an affected father (65 years) [8]. Similarly, another retrospective analysis found that age of ESRD was earlier in males who inherited disease from the mother compared to the father (46 vs. 54 years) [9], in contrast to our findings. However, more consistent with the current study, an analysis of 296 French individuals with ADPKD found mean age at ESRD was younger with fathers transmitting ADPKD (52 years) compared to mothers (61 years) [10]. 

To our knowledge, while the effect of parental hypertension status on hypertension in an offspring has previously been evaluated [22], the effect of parental sex upon age of development of hypertension has not been previously evaluated. Similar to the ESRD endpoint, we found a difference in time to hypertension according to parental sex only in male participants, with hypertension occurring earlier in males with an affected father compared to an affected mother. These results are in contrast to the concept of an adverse in-utero environment from an affected mother effecting later disease progression. 

However, the earlier median age of death in participants with an affected mother compared to an affected father is inconsistent with the ESRD and hypertension endpoints. One previous analysis of changes in mortality ratios across a century-long analysis of five ADPKD families found no evidence of either participant sex or parent sex influences upon mortality [23]. Our findings appear to have been driven in part by four deaths that occurred either in utero or during infancy in participants with an affected mother. Sensitivity analyses excluding these individuals attenuated the difference between groups, but median age of death still occurred earlier in participants with an affected mother compared to an affected father, thus early deaths cannot fully account for these group differences, and other non-cardiovascular and non-ESRD causes of death likely contributed. Of note, these early deaths occurred in the presence of severe cystic disease and in families with a known *PKD1* genotype; however, complex mutations involving additional genes may have modified the phenotype. A possible explanation of severity of disease is coinheritance of a hypomorphic allele from the nonaffected parent [24]. Similarly, we observed that participants with an affected mother were more likely to have VEO disease than participants with an affected father. This result is consistent with some [25, 26, 27], but not all, previous [17, 28] studies examining the influence of parental sex upon VEO disease, and does support the possibility of genetic imprinting or an adverse in-utero environment. 

We decided a priori to perform stratified analyses according to participant sex in addition to parental sex due to a large number of studies indicating that median age at ESRD is younger in males compared to females [9, 11, 20, 21]. However, this finding has not been universal [8, 10, 29], and one prior analysis suggested a potential interaction between participant and parental sex [9]. Our results indicate that the combined influences of participant and parental sex may indeed be important in predicting severity of ADPKD, as time to both hypertension and ESRD were earlier in males (compared to females) with an affected father, but not an affected mother. Previous studies have not evaluated the influence of parental sex or the interaction of participant and parental sex upon the development of hypertension in ADPKD patients. Prevalence of hypertension is greater and age of onset is slightly earlier in males compared to females with APDKD [22, 30]. 

Our finding that hypertension and ESRD occurred earlier in males with an affected father, but not an affected mother, are in contrast to our hypothesis. However, genetic imprinting may still occur, with an effect from the father rather than the mother. The concept of genetic imprinting proposes that there is differential modification of genetic material (DNA marks) depending on whether inheritance is from the mother or the father [15]. The limited number of studies in rodents [31] and humans [8, 32] that have suggested genetic imprinting have indicated a role of maternal rather than paternal inheritance of ADPKD. However, the greater prevalence of VEO status in participants with affected mothers compared to affected fathers suggests that the in-utero environment may possibly influence disease severity, consistent with the concept that the prenatal environment can induce epigenetic modification that influence phenotype and disease risk [16]. Alternatively, compound mutations may have contributed to the VEO phenotype, and further genotyping would be required to elucidate contributing mechanisms. Additionally, longer follow-up of those with VEO-ADPKD will be necessary to fully evaluate disease severity and progression in this subset of patients. 

There are several limitations to this analysis. As the study was retrospective, various biases are introduced including selection bias, loss to follow-up, and missing data. Many endpoints were self-reported, which may have resulted in misclassification bias, and information on all endpoints was missing for some participants. Additionally, due to the nature of self-reported follow-up in many participants, additional outcomes, such as change in estimated glomerular filtration rate, were not available for analysis. As this is an observational study design, it only shows association rather than causation, and the cohort is from a single center. While all participants included had the presence of the *PKD1* genotype, determined by linkage analysis, detailed information on mutation type was unavailable in this cohort and represents an important future direction. These findings may differ in individuals with the *PKD2* genotype, and future research should follow up on previous research in this population [11]. Potential bias may exist regarding prevalence of VEO status if mothers with ADPKD were more likely to have received a prenatal ultrasound than an unaffected mother; however, this is unlikely to be the case. Additionally, cause of death was unavailable in ~ 30% of participants. 

The major strength of this analysis is that it included a relatively large number of participants with ADPKD with longitudinal data collected over a nearly 30-year period. While most previous analyses have examined only an ESRD endpoint, we additionally examined the age at diagnosis of hypertension as well as age of death. Additionally, most previous analyses have examined the role of participant rather than parental sex, and little literature is available on the interaction between participant and parental sex. 

Our results are largely in contrast to the hypothesis that severity of ADPKD is worse with maternal inheritance of disease. With the exception of mortality, which may or may not be ADPKD-related, adverse outcomes (hypertension and ESRD) occurred earlier in males than females, but only with paternal inheritance. However, maternal inheritance was associated with a greater likelihood of VEO disease as well as deaths in utero or during infancy. Overall, these findings support the concept that while genetic imprinting and/or in-utero environment may play a role in disease severity, other factors, including type of mutation [5] or other genes that modify disease severity [7] as well as gene-environment interactions [6], are likely to be greater contributors to the variability seen in the course of progression of ADPKD [5, 6]. 

## Funding 

This work was supported by National Institutes of Health awards (DK103678, DK034039, and UL1 TR001082). 

## Conflict of interest 

Berenice Gitomer and Michel Chonchol have previously received research funding from Otsuka, which did not support the current study. 

**Table 1.** Clinical characteristics of ADPKD study participants according to sex of affected parent.

Variable	Affected parent = mother (n = 436)	Affected parent = father (n = 378)	p-value
Age at diagnosis (years)	23 ± 15	26 ± 13	0.04
Sex (n, % female)	237 (54.4%)	206 (54.5%)	0.97
History of hypertension n, (%)	197 (67.2%)	205 (73.5%)	0.10
Reached ESRD (n, %)	133 (31.1%)	138 (37.4%)	0.06
Died (n, %)	124 (28.4%)	107 (28.3%)	0.97
Very-early onset ADPKD (n, %)	32 (7.3%)	5 (1.3%)	< 0.0001
Cause of death			0.38
Cardiovascular (n, %)	25 (20.2%)	24 (22.4%)	
Neurological event (n, %)	18 (14.5%)	14 (13.1%)	
ESRD (n, %)	12 (9.7%)	16 (15.0%)	
Infection (n, %)	11 (8.9%)	7 (6.5%)	
Cancer (n, %)	7 (5.6%)	7 (6.5%)	
Trauma (n, %)	3 (2.4%)	6 (5.6%)	
Gastrointestinal (n, %)	3 (2.4%)	1 (0.8%)	
In utero or infancy (n, %)	4 (3.2%)	0 (0.0%)	
Pulmonary (n, %)	3 (1.3%)	0 (0.0%)	
Other/Not reported (n, %)	38 (30.7%)	32 (29.9%)	

Data are mean ± SD or n (%) for those without missing data. Data are missing for age of diagnosis (n = 341), history of hypertension (n = 143), and reached end-stage renal disease (ESRD; n = 8). Cause of death is only reported for those who died (n = 231). ESRD as a cause of death represents known withdrawal from or refusal of dialysis. See methods for “other” causes of death. ADPKD = autosomal dominant polycystic kidney disease; ESRD = end-stage renal disease.

**Table 2.** Clinical characteristics of autosomal dominant polycystic kidney disease (ADPKD) study participants according to sex of affected parent and further stratified by patient sex.

Variable	Affected parent = mother (n = 477)	Affected parent = father (n = 420)	p-value
Median age of hypertension (years)	35 (27, 43)	35 (27, 41)	0.97
Median age at ESRD (years)	56 (49, 61)	55 (48, 62)	0.79
Median age of death (years)	56 (49, 64)	60 (51, 66)	0.02
Stratified			
Age at diagnosis for males (years)	25 ± 15	26 ± 13	0.09
Age at diagnosis for females (years)	22 ± 15	25 ± 12	
Age of hypertension for males (years)	33 (25, 41)	32 (24, 38)	0.004
Age of hypertension for females (years)	37 (28, 44)	36 (30, 44)	
Age of ESRD for males (years)	54 (48, 60)	52 (47, 59)	< 0.001
Age of ESRD for females (years)	58 (51, 65)	60 (51, 65)	
Age of death for males (years)	55 (48, 63)	57 (50, 64)	0.01
Age of death for females (years)	58 (51, 64)	61 (53, 67)	

Data are median (IQR) or mean ± SD for those without missing data. p-values are for the log-rank test (except age of diagnosis (ANOVA)). Data are missing for hypertension (n = 143) and end-stage renal disease (ESRD; n = 8). Sidak adjusted (for multiple comparisons of log-rank test) p = 0.008 for age of hypertension between males and females when the affected parent is the father and p = 0.14 for age of hypertension between males and females when the affected parent is the mother. Sidak adjusted p = 0.0008 for age of ESRD between males and females when the affected parent is the father and p = 0.11 for age of ESRD between males and females when the affected parent is the mother. Sidak adjusted p = 0.07 for age of death between males and females when the affected parent is the father and p = 0.50 for age of death between males and females when the affected parent is the mother. ADPKD = autosomal dominant polycystic kidney disease; ESRD = end-stage renal disease.
